# The Role of Thyroglobulin in Preoperative and Postoperative Evaluation of Patients With Differentiated Thyroid Cancer

**DOI:** 10.3389/fendo.2022.872527

**Published:** 2022-06-02

**Authors:** Sha Li, Chutong Ren, Yi Gong, Fei Ye, Yulong Tang, Jiangyue Xu, Can Guo, Jiangsheng Huang

**Affiliations:** Department of Thyroid Surgery, The Second Xiangya Hospital of Central South University, Changsha, China

**Keywords:** thyroglobulin, differentiated thyroid carcinoma, preoperative evaluation, postoperative evaluation, role

## Abstract

Thyroglobulin (Tg) is secreted by thyroid follicular cells and stored in the thyroid follicular lumen as a component of thyroid hormone. It is known that both benign and well-differentiated malignant thyroid tissue can secrete Tg. In recent years, growing lines of evidence have shown that Tg plays an important role in the diagnosis and metastasis of preoperative differentiated thyroid carcinoma (DTC). The levels of Tg, whether in the serum or in a fine-needle aspiration washout fluid, are usually viewed as an excellent indicator in the monitoring of postoperative DTC, including the guidance and evaluation of radioactive iodine ablation. Nevertheless, some factors limit the application of Tg, such as the method used to measure Tg and the presence of Tg antibodies. This review aimed to summarize the role of Tg in the preoperative and postoperative evaluation of patients with DTC, and the factors influencing Tg. This review could provide a reference for a more accurate application of Tg in patients with DTC.

## 1 Introduction

Thyroid cancer, the most common endocrine malignant tumor, has shown an increasing incidence rate, ranking ninth in 2020 globally (1). It is predicted that thyroid cancer will be the fourth most common cancer in 2030 (2). Owing to the stable incidence of other pathological types of thyroid cancer, the increasing tendency noted above occurs with differentiated thyroid carcinoma (DTC), and DTC accounts for the greatest proportion of thyroid cancer (3, 4). At present, owing to the extensive use of clinical imaging and the deep awareness of the importance of health examinations, more than 68% of the general population have been diagnosed with thyroid nodules (TNs) (5). The preliminary screening of thyroid nodules includes thyroid ultrasound, which is crucial for the identification of benign and malignant TNs, contrast-enhanced ultrasound, and ultrasonic elastography used for further diagnosis. Ultrasound-guided fine needle aspiration (FNA) is the most effective method for the diagnosis of DTC before surgery (6). However, nodule size, features, and clinicians’ experience may affect the accuracy of ultrasound and FNA (7). Moreover, results are uncertain in 15%–20% of thyroid cytology and a quarter of these nodules are malignant (8). Therefore, predictive factors are particularly important for identifying benign and malignant TNs. Molecular biomarkers are used for the evaluation of TNs, including RET/PTC, BRAF, and RAS. Preoperative serum Tg is also useful (9). Apart from the diagnosis of TNs, accurate and effective prognostic evaluation is also vital for thyroid cancer. Some methods are used for follow-up after thyroidectomy and the formulation of treatment plans, including ultrasound and the serum thyroid function and thyroglobulin (Tg) (10).

**Figure 1:**
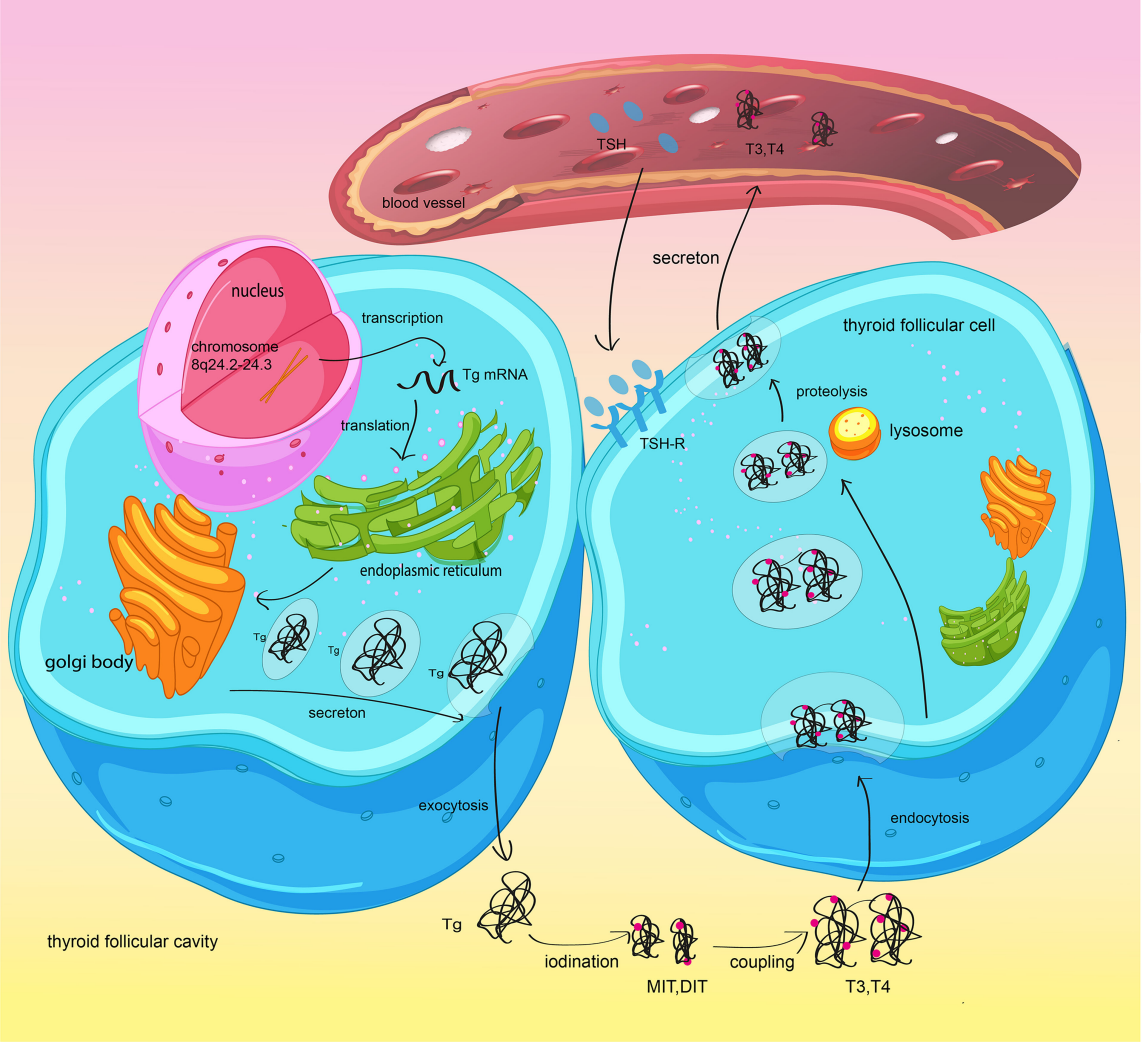
Synthesis and secretion of thyroglobulin and thyroid hormones. Thyroid follicular cells synthetize Tg and secrete it into the thyroid follicular lumen by exocytosis. Reactive iodide is covalently linked to specific tyrosyl residues of thyroglobulin (Tg) and generate MIT and/or DIT. MIT/DIT form T3 and T4 through coupling. When TSH combines with TSH-R in thyroid follicular cells, the newly synthesized thyroid hormone will be increasingly transported to the bloodstream.

Tg is a large protein dimer of 660 kDa produced by thyroid follicular cells and is integral to thyroid hormone synthesis. In this process, tyrosine residues of Tg are iodinated to form monoiodotyrosines and diiodotyrosines. Iodinated Tg is stored in the thyroid follicular lumen, and its release is influenced by thyroid stimulating hormone (TSH). Subsequently, these iodotyrosines undergo oxidative coupling to form T3 and T4 (**Figure 1**). Some researchers have shown that Tg gene mutation can facilitate the development of thyroid cancer by obstructing thyroid hormone synthesis (11), highlighting the significance of Tg in the identification of malignant thyroid nodules. Tg is produced by both benign and well-differentiated malignant thyroid cells, which means that it marks the existence of thyroid tissue (12, 13). Consequently, the level of Tg after thyroidectomy for thyroid cancer is used to predict tumor recurrence and metastasis. However, a recent study found that some methods of Tg measurement may not accurately reflect serum Tg *in vivo*. The existence of Tg antibodies (TgAb) might reduce the level of Tg and result in a false-negative test. These factors limited the application of Tg in DTC.

**Figure 2:**
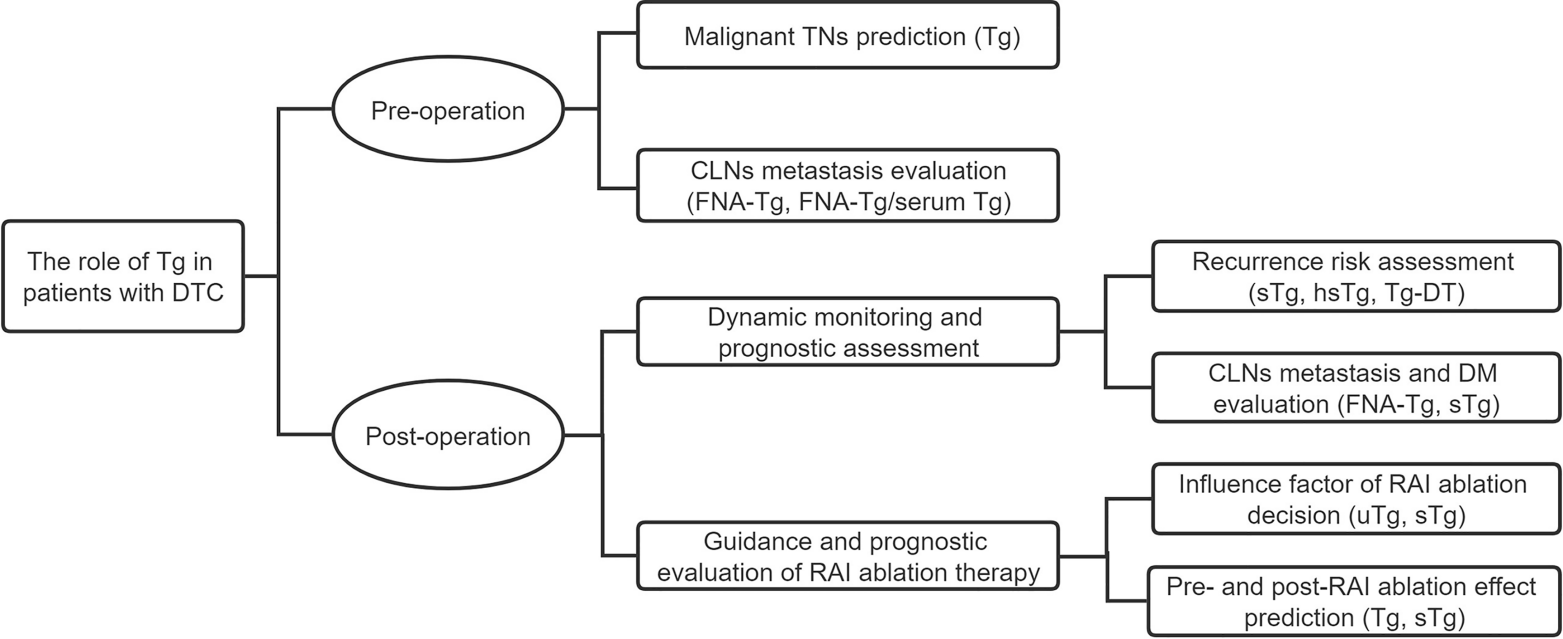
Outline of the role of thyroglobulin in patients with differentiated thyroid cancer.

In this review, we summarize the role of Tg in DTC, including preoperative prediction and postoperative evaluation and the factors that influence Tg levels (**Figure 2**). We comprehensively expound Tg measurement and the best applications to be used in different situations. References are provided for the accurate preoperative evaluation of Tg, the prognostic prediction of DTC, and the selection of the Tg measuring method.

## 2 The Role of Tg in Preoperative Patients With DTC

### 2.1 The Evaluation Value of Tg in Preoperative DTC

Preoperative serum Tg evaluation of patients with DTC remains controversial in the assessment of TNs. Because of deviations in Tg in thyroid disease, some early studies suggest against the routine measurement of serum Tg levels, considering it to be a hyposensitive and non-specific test (14). The level of serum Tg is closely associated with the total mass of the thyroid *in vivo*, thyroid stimulation induced by TSH, and invasive surgeries such as FNA (15). For instance, when benign TNs are complicated by goiter and thyroiditis, preoperative Tg also rises owing to an increase in the number of follicular cells or damage to follicular cells (16). Giovanella et al. (17) stated that preoperative serum Tg was undetectable or reduced among 3.0%–5.1% of patients with DTC, by whatever means. In contrast, recent studies considered serum Tg as a risk factor and an independent predictor for thyroid cancer. Kars et al. (18) found that the prevalence rate of thyroid cancer was higher with preoperative serum Tg >188.5 ng/ml. Wang et al. (19) showed that an increasing ratio of preoperative serum TSH and Tg was more relevant to thyroid cancer compared with TSH alone, and the ratio was viewed as a risk factor for malignancy. Scheffler et al. (20) produced the McGill Thyroid Nodule Score + (MTNS+) and found that when a preoperative serum Tg value was 75 ng/ml, it added one point to the MTNS; whereas a Tg value of 187.5 ng/ml added two points. They showed that the practical use of MTNS+ was superior to that of MTNS because of Tg. Furthermore, MTNS+ could produce an accurate risk stratification of highly differentiated malignant tumors of the thyroid gland. Preoperative Tg is also helpful for predicting cervical lymph node (CLN) metastasis and choice of surgery (21). In the same year, Huang et al. (22) initially proposed that Tg was related to skip metastasis of papillary thyroid carcinoma (PTC). Preoperative serum Tg was verified as an effective predictor in initial distant metastasis (DM) of DTC (23). A systematic review showed that preoperative serum Tg should be measured, especially along with uncertain cytology (24). At present, we see preoperative serum Tg as a reference to predict malignant TNs. In general, further research, including prospective studies, regarding the value of preoperative serum Tg in thyroid cancer is still needed.

### 2.2 The Role of Tg Measurement With FNA-Tg in Preoperative DTC

FNA-Tg is the measurement of Tg level in the washout fluid from FNA. Notably, many recent studies have verified that FNA-Tg was helpful for evaluating CLN metastasis. In 1992, FNA-Tg was firstly shown by Pacini et al. (25) to indicate CLN metastasis of DTC. Subsequently, several studies have proposed that FNA-Tg could be used for preoperative CLN detection in DTC (26). Uruno et al. (27) considered CLNs with FNA-Tg levels higher than serum Tg levels as positive. The results showed that the sensitivity of FNA-Tg was 81.4%, which was higher than the 78.0% of fine-needle aspiration cytology (FNA-C). It was concluded that FNA-Tg was an effective method for the diagnosis of preoperative CLN metastasis in PTC. Al-Hilli et al. (28) also confirmed that the sensitivity of FNA-Tg was superior to that of FNA-C and using the two methods together could improve the detection efficiency of CLN metastases by 13%. In 2012, Kim et al. (29) obtained FNA-Tg levels within suspected metastatic CLNs in PTC. FNA-Tg concentrations of >50 ng/ml were viewed as positive. The authors suggested that FNA-Tg could be used to assess CLN metastasis of PTC and that its diagnostic performance was not influenced by the existence of thyroid gland tissue, which suggested the reliability of FNA-Tg for the evaluation of preoperative CLNs of PTC. However, the cutoff value of FNA-Tg has not been informative. Pak et al. (30) suggested that when FNA-Tg was >32.04 ng/ml, it indicated CLN metastasis. In the same year, Jeon et al. (31) found that the cutoff value of FNA-Tg was related to the level of serum Tg. When serum Tg value was ≤1.0 μg/L, they recommended viewing 1.0 μg/L as the cutoff value of FNA-Tg. However, when serum Tg was >1.0 μg/L, a ratio of 0.5, which was the level of FNA-Tg divided by the level of serum Tg, showed better performance. Later, Liu et al. (32) demonstrated that FNA-C and FNA-Tg/serum-Tg performed together showed higher specificity when compared with combined FNA-C and FNA-Tg. Furthermore, they found that multilevel FNA-Tg was also helpful in identifying lateral CLN metastasis. In summary, although the cutoff value of FNA-Tg has remained controversial, the value of FNA-Tg and FNA-Tg value/serum-Tg is acknowledged by most authors.

## 3 The Role of Tg in Postoperative Patients With DTC

### 3.1 The Prognostic Role of Tg in Postoperative Patients With DTC

Due to the excellent curative ratio of DTC after thyroidectomy and the adjuvant administration of radioactive iodine (RAI) ablation, most postoperative patients have no obvious clinical symptoms at follow-up. Therefore, there needs to be highly specific and sensitive monitoring methods for long-term follow-up so that recurrent patients can receive timely and effective intervention and patients without recurrent cancer can avoid unnecessary tests and treatments (33). The 2015 American Thyroid Association (ATA) management guidelines proposed the measurement of Tg as a valid way to dynamically monitor the prognosis of DTC. It was recommended that a stimulated Tg (sTg) of <0.2 ng/ml or 0.1 ng/ml was used for the cutoff to evaluate DTC recurrence (34). In the past several years, a series of studies verified the prognostic evaluation role of Tg. Durante et al. (35) found that the serum Tg level of most patients without RAI ablation dropped down to undetectable level during 5–7 years after thyroidectomy, meaning that once serum Tg level went up, it might forebode a poor prognosis of DTC. Besides serum Tg, there are also other Tg types used in the monitoring of DTC (**Table 1**).

**Table 1:** Evaluation forms of thyroglobulin in the monitoring of differentiated thyroid cancer.

Evaluation forms	Definition
Tg measurement with fine-needle aspiration (FNA-Tg)	Tg concentrations of washout fluid from FNA
Stimulated-Tg (sTg)	Serum Tg levels after adopting recombinant human TSH (rhTSH) or thyroid hormone withdrawal
Unstimulated Tg (uTg)	Serum Tg levels without TSH stimulation
High-sensitive Tg (hsTg)	Function sensitivity of 0.1–0.2 μg/L
Tg doubling time (Tg-DT)	The time of twice Tg concentration

Stimulated serum Tg by recombinant human TSH (rhTSH) or thyroid hormone withdrawal was initially used for prognostic evaluation. The ATA strongly recommended that high-risk patients should undergo a serum Tg test every 6–12 months. A study reported that sTg <1 ng/ml in high-risk PTC patients indicated a good prognosis (36). Jayasekara et al. (37) suggested that measuring sTg during the early postoperative period could accurately quantify the risk of DTC recurrence. A retrospective study measured rhTSH-stimulated Tg level again after an initial undetectable rhTSH-stimulated Tg level. It drew a conclusion that the initial low or negative rhTSH-stimulated Tg level was a good indicator for slow disease progression and the second negative rhTSH-stimulated Tg level suggested a reassuring result. Under these circumstances, postoperative follow-up could be performed by a nonstimulated Tg every few years (38). Another retrospective study measured serum Tg after thyroxine withdrawal for 4 weeks (LT4 withdraw) to evaluate the prognosis of recurrence after reoperation. It suggested that recrudescent patients should routinely be measured for Tg after LT4 withdrawal, particularly when it was >10 ng/ml. Tg after LT4 withdrawal was an excellent indicator for supervision recurrence after reoperation (39). The latest study found that high-sensitive Tg (hsTg) measurement could greatly simplify postoperative management of DTC by avoiding stimulation (40, 41). Malandrino et al. (42) confirmed that hsTg could identify patient disease progression, and in most patients, it was sufficient to measure hsTg. When hsTg level was ≥1 μg/L or increased with time in TgAb-negative patients, a neck ultrasound was recommended (43).

When serum Tg is used as the indicator to evaluate residual thyroid tissue, some factors influence the accuracy, such as the concentration of TSH and the existence of TgAb (15). Some researchers consider the change of serum Tg with time after thyroidectomy, especially the Tg doubling time (DT). Multivariate analysis indicated that Tg-DT was not only an independent prognostic factor of survival, distant metastasis, and local recurrence, but also a more accurate prognostic predictor compared with other classical predictors including TNM stage, age, and gender (44). Rössing et al. (45) evaluated Tg-DT and other prognostic factors by uni- and multivariate analysis in progressing DTC. They found that the mortality risk with Tg-DT >14 months was twofold lower than Tg-DT <5 months. The study indicated that Tg-DT was not an independent predictor of survival in progressive DTC. In summary, most authors support the significance of Tg-DT for dynamic risk assessment in postoperative patients.

Postoperative positive Tg is related to CLN metastasis and DM. A series of studies have shown that the Tg test in FNA of CLNs is an effective way to identify CLN metastasis after thyroidectomy. In a study by Cunha et al. (46), the sensitivity of FNA-Tg was 100% in early CLN recurrence. Even if TgAb were present, it enhanced the accuracy of cytologic examination (47). A retrospective study showed that FNA-Tg measurement might replace CLN cytologic examination (48). Pacini et al. (25) suggested that improvement of FNA-Tg in CLNs was indicative of CLN metastasis from DTC. The cutoff of FNA-Tg in CLNs does not have a standard. Borel et al. (49) verified that low FNA-Tg levels of CLNs were irrelevant to serum Tg levels and could imply metastasis of DTC. Jeon et al. (30) recommended FNA-Tg in CLNs of 0.9 ng/ml as the cutoff to identify CLN metastasis. A further study measured FNA-Tg level in lung metastasis guided by CT and determined that it was an effective method and also reduced the number of invasive operations at the same time (50). In addition to FNA-Tg, serum Tg showed a certain value for predicting DM of DTC (51). Demir et al. (52) used serum Tg and Tg/TSH values associated with DM and found that their areas under ROC were 0.990 and 0.991, respectively. Tg/TSH was therefore confirmed as an early biomarker for DM (53). A later study demonstrated that sTg levels >117.5 ng/ml indicated DM in DTC, whose negative predictive value was up to 93.7% (54). Particularly in children and adolescent patients with DTC, sTg showed a higher accuracy in predicting DM (55, 56).

Apart from the above-mentioned papers, some researchers have also tried to study the role of serum Tg in DTC lobectomy. Some authors consider that after lobectomy, patients still have a normal thyroid gland, and it is not possible to identify whether improvement of serum Tg comes from normal or recurrent tissue. A series of studies showed that there was limited value for serum Tg measurement after lobectomy to identify local recurrence (57, 58). Tourani et al. (59) even found that serum Tg was useless for the management of patients following a lobectomy.

Tg measurement, neck ultrasound, and ^131^I whole-body scan (WBS) are the primary assessment methods for follow-up patients with DTC (60). Because of the complex technology needed and the high testing cost, serum Tg value and neck ultrasound have gradually replaced ^131^I WBS in low- and medium-risk patients, and they provide a credible postoperative evaluation (61, 62). Fernandes et al. (63) stated that Tg measurement and neck ultrasound should be recommended 6 and 12 months postoperatively, and then annually if the results are negative. During a 1-year evaluation, low- and medium-risk patients with negative neck ultrasound and Tg level <1 ng/ml could be monitored solely by unstimulated serum Tg and clinical assessments (64, 65). Neck ultrasound was suggested when the Tg level trend was increasing (66, 67). The ATA and NCCN guidelines recommend repeated Tg measurement with rhTSH stimulation when the initial Tg value is undetectable. Some researchers showed that rhTSH-stimulated Tg value did not change the postoperative management if suppressed Tg was <1 ng/ml and neck ultrasound was negative (68, 69). Castagna et al. (70) proposed that rhTSH-stimulated Tg should be repeatedly measured at a first positive rhTSH-stimulated Tg and negative neck ultrasound. For most low- and medium-risk patients, suppressed Tg measurement and neck ultrasound are sufficient to predict recurrence of DTC. However, if some indicators are positive, it is crucial to perform further tests.

### 3.2 The Role of Tg in Postoperative RAI Ablation

As early as the 1970s, RAI had been viewed as the best method of remnant ablation after thyroidectomy. At that time, RAI ablation therapy was limited in patients with poor prognosis, because the heritability and carcinogenicity of RAI caused panic (71). Nevertheless, a report by Mazzaferri et al. (72) found that RAI ablation could improve overall survival and reduce recurrence rate. Since the 1980s, when residual thyroid malignant tissue after thyroid surgery became common, RAI remnant ablation has been recommended for most patients with DTC. However, RAI remnant ablation in low-risk patients does not seem to have much value compared with its side effects and treatment costs. Current guidelines recommend that RAI ablation should be used more selectively, instead of as a routine, and administration dose should be lower. Currently, there needs to be further clarification regarding the individual use of RAI ablation and the number of doses. Tg was considered as a strong prognostic factor in DTC. There have also been many reports that have studied its influence on RAI ablation. Next, we summarize the role of Tg for RAI ablation with the aim of suggesting protocols for RAI ablation therapy.

#### 3.2.1 Guidance Regarding the Role of Tg Before RAI Ablation

In recent years, increasing number of scholars consider that serum Tg after thyroidectomy is an indicator guiding the use of RAI ablation. As Ibrahimpasic et al. (73) reported, it is safe for low- and moderate-risk patients with DTC after total thyroidectomy not to receive RAI ablation therapy, when their serum Tg is undetectable. Similarly, Mourão et al. (74) found that it was unnecessary to perform RAI ablation in postoperative patients with low unstimulated Tg [uTg (uTg <0.3 ng/ml)] and a negative neck ultrasound. In the same year, a different cutoff of uTg was proposed by Rosario et al. (75). They suggested that with low uTg (<0.25 ng/ml) and negative TgAb and neck ultrasound, low-risk patients with PTC whose tumor size was >1 cm should not undergo RAI ablation. However, what about low-risk patients with a tumor size >4 cm? Mourão et al. (74) had followed up patients with PTC >4 cm for 5 years, whose sTg was ≤2 ng/ml or uTg <0.3 ng/ml with negative imaging results. None of these patients received RAI ablation, and no structural recurrence was found during the 5-year follow-up. In a series of studies by Orlov et al. (76), all patients without RAI ablation (including those with nidus >4 cm) showed no recurrence, when their postoperative serum sTg values were <5 ng/ml. They allowed patients with undetected sTg or an sTg of 1–5 ng/ml to not receive RAI ablation, while patients with sTg >5 ng/ml received RAI ablation. Thus, those patients with undetected sTg or sTg 1–5 ng/ml whose tumors were >4 cm, whose ages were greater than 45 years, and whose CNLs were involved avoided RAI overtreatment. There were no recurrent cases in patients without RAI ablation and only patients receiving RAI ablation showed recurrent clues. More importantly, sTg seemed to be helpful in determining the dose of RAI ablation. Nonmetastatic patients with sTg <5 ng/ml receiving low-dose RAI had a similar outcome to those receiving high-dose RAI (77). Therefore, we suggest that low postoperative sTg or uTg might be one of the standards to address the unnecessary treatment of RAI ablation. The cutoff of sTg and uTg still needs to be further determined (78).

Postoperative sTg can both guide RAI ablation treatment and predict the curative effect before RAI ablation. Some studies showed that patients with low sTg and limited LN metastasis after total thyroidectomy tended to be cured after the first RAI ablation therapy (79). Spaas et al. (80) determined that an sTg value before RAI ablation was a good indicator of recrudescence of DTC. Measurement of serum Tg on the first day and third day of rhTSH administration (Tg1; Tg3) was performed by Ledwon et al. (81). They identified that the cutoff of Tg1 and Tg3, respectively, was 0.7 ng/ml and 1.4 ng/ml, which were able to independently predict RAI ablation success in DTC patients with total or near-total thyroidectomy. Besides sTg, the data analysis of Trevizam et al. (82) indicated that the sTg/TSH ratio was as reliable as sTg in the prediction of the RAI ablation effect. Prpic et al. (83) evaluated 740 patients and determined Tg/TSH to be a more reliable factor compared with serum Tg alone. In multivariable logistic regression analysis, elevated Tg/TSH was identified as an independent prognostic factor for RAI ablation. Based on the proposition of Tg/TSH, further studies are needed to verify the predictive value at the time of TSH stimulation.

In summary, postoperative serum Tg is a significant factor in the decision to perform RAI ablation therapy and prediction of the RAI ablation effect. Of note, serum Tg is not the only indicator for evaluation of RAI ablation treatment. Moreover, the 2015 ATA guidance does not have a specific value of serum Tg to guide and predict ARI ablation (34). More importantly, with increasing numbers of thorough studies, the ratio of Tg/TSH has gradually gained importance as a predictive indicator in RAI ablation.

#### 3.2.2 The Prognostic Role of Tg After RAI Ablation

After RAI ablation therapy, the level of Tg similarly has a prognostic and instructional function. Initially, one thing must be understood: the level of serum Tg rises transitorily during an early stage of post-RAI ablation in DTC patients with total thyroidectomy. Destruction and inflammation of thyroid tissue by RAI reflection induce the release of Tg in residual thyroid tissue, which might lead to the transient rise of Tg. This value will not drop below baseline for approximately 6 months (84). Frank et al. (85) stated that the level of Tg would slightly increase after RAI ablation in total thyroidectomy, and they recommend sequential surveillance and follow-up instead of additional RAI ablation. Moreover, the increase of Tg after 2 days following RAI ablation might predict a good result in DTC patients with distant metastasis, which indicates that more thyroid tissue was destroyed (86). Accordingly, we concluded that Tg after RAI ablation should be measured at least 6 months later to predict the effect of RAI ablation and guide subsequent therapy. The level of Tg in most patients with RAI ablation was very low or undetected. Padovani et al. (87) suggested that patients with DTC might only need close follow-up when the Tg value at 6 months of RAI ablation dropped to 1–5 ng/ml. In this situation, neck US became an unnecessary examination during the period of follow-up. Neck US was only performed with Tg values of >1.0 μg/L (43). In WBS-positive and Tg-negative patients (WBS+Tg-), Lim et al. (88) proposed that the best treatment option might be continued observation without repeated RAI ablation. A study by Piccardo et al. (89) found that a Tg level of ≥50 μg/L was a valuable cutoff to predict recrudescence in high-risk patients with DTC. Afterward, Wong et al. (90) used ROC analysis of sensitivities and specificities and suggested a 1 ng/ml cutoff of sTg after ARI ablation at around 6 months to predict adverse clinical results. In contrast, it has been reported that a patient with an extremely low level of Tg after RAI ablation developed distant metastases in the brain and lungs, even when his WBS was negative. This report implied that it seemed unsafe to view Tg level or WBS as a single marker (91). Notably, the level of Tg after the first RAI ablation was also regarded as an effective decisive and prognostic indicator for the second RAI ablation (92). Abe et al. (93) proposed that patients with a serum Tg >9 ng/ml after the first RAI ablation might need a subsequent higher dose of RAI. Recently, Huang et al. (94) found a new biomarker in thyroid cancer named urinary exosomal thyroglobulin (U-Ex Tg), which may replace serum Tg in the future. In their study, U-Ex Tg was high in patients with CLN metastasis and lymph-vascular invasion. Additionally, serum Tg was undetectable after RAI ablation while the rising tendency of U-Ex Tg implied possible recurrence.

## 4 Factors Influencing Tg Level

Serum Tg concentration is influenced by many factors. Full details are given in **Table 2**. In this section, we focus on the influence of the methods used to measure Tg and TgAb.

**Table 2:** Factors influencing serum thyroglobulin levels in patients with differentiated thyroid cancer.

Influence factors	Examples
Amount of residual thyroid tissue	Differentiated thyroid carcinoma tissue and normal thyroid tissue
Invasive manipulation of thyroid gland	FNA, RAI ablation, and surgery
Other concomitant thyroid diseases	Thyroiditis like Hashimoto thyroiditis (HT)
TSH receptor stimulation	The serum TSH levels, rhTSH, and thyrotropin receptor antibody (TRAb)
Tg measuring methods	IMA, RIA, and LC-MS/MS
Thyroglobulin antibody (TgAb)	——

### 4.1 The Influence of Measuring Methods on Tg Level

Tg measurements include immunometric assays (IMA), radioimmunoassay (RIA), and liquid chromatography/tandem mass spectrometry (LC-MS/MS) assays. Tg measured by IMA is the most common method used in clinical laboratories. Essentially, it is a two-site reaction involving a solid-phase antibody and a labeled antibody. The second-generation IMA with a functional sensitivity (FS) of <0.2 ng/ml gradually replaced the first-generation IMA with an FS of 1 ng/ml, which reduced the need for TSH stimulation (95). For patients with positive TgAb, IMA might not be satisfactory. Because of the interference of TgAb, the serum Tg measured by IMA might be fictitiously reduced, even when the level of TgAb is low or lower than the cutoff (96). Apart from TgAb, the presence of heterophile antibodies (HAb) might result in high Tg measured by interacting with antibodies in IMA (97).

Tg measured by RIA indicates that serum Tg is combined with a limited amount of a high-affinity rabbit polyclonal TgAb competing with a radiolabeled (^125^I) human Tg (98, 99). The FS of RIA ranges from 5 to 15 μg/L (100). Currently, because of the need to handle radioactive materials, RIA has not been widely applied. However, it is extensively used to evaluate the interference of TgAb, though its FS remains unsatisfactory; its polyclonal antibodies binding with TgAb can still recognize Tg epitopes (98). Compared with IMA, the interference of TgAb seems lower than that for Tg levels by RIA. A study tested Tg levels, respectively, using IMA and RIA in normal thyroid subjects with positive and negative TgAb. Tg levels measured by RIA with negative TgAb were approximately the same as Tg levels with positive TgAb. However, Tg levels measured by IMA with positive TgAb were generally lower or even undetectable.

Tg measured by LC-MS/MS uses tryptic digestion and immunocapture to overcome TgAb interference, because all proteins, including TgAb and HAb, can be dissociated (101). The technique belongs to a new and cost-effective technology that could be used for Tg measurement in the presence of TgAb (102). The operation and maintenance of instrumentation and sample throughput limit its application to a certain extent (103). The levels of Tg with positive TgAb as detected by LC-MS/MS are commonly higher than the levels of Tg as detected by IMA (101). LC-MS/MS assays can identify serum Tg in the presence of TgAb when IMA cannot. Kushnir et al. (102) verified that 23% of positive TgAb samples could not detect Tg concentrations by IMA, while LC-MS/MS assay results gave Tg concentrations from 0.7 to 11 μg/L.

In summary, Tg measurements have advantages and disadvantages (**Table 3**). Currently, the IMAs are the most commonly used methods in clinical laboratories. It is known that IMAs always underestimate Tg levels in the presence of TgAb (104). RIA and LC-MS/MS could overcome the interference of TgAb. However, Tg measured by RIA can sometimes be falsely low or falsely high; the FS of RIA can be lower than the second-generation IMA. Crane et al. (105) proposed that Tg measured by RIA and IMA together should be viewed as a feasible monitoring method in the presence of TgAb. As for LC-MS/MS, more clinical trials are needed to verify the performance of Tg measurement in patients with positive TgAb and structural disease.

**Table 3:** Thyroglobulin measuring methods in patients with differentiated thyroid cancer.

Methods	Advantages	Disadvantages
Immunometric assays (IMA)	Application in most clinical laboratories Simple operation steps Short turnaround times Pretty functional sensitivity.	High susceptibility to TgAb and HAb interference. (falsely low results)
Radioimmunoassay (RIA)	Resistance to TgAb interference	Unsatisfactory functional sensitivity Positive bias
Liquid chromatography/tandem mass spectrometry (LC-MS/MS) assays	Elimination of TgAb and HAb interference	Increased testing costs Unsatisfactory functional sensitivity Undetectable Tg in structural diseases Potential differences in calibration

### 4.2 The Interference of TgAb With Tg Level

TgAb is the most important interfering factor in the measurement of Tg level. To some extent, it limits the application of Tg testing in DTC. About 20% of patients with DTC have detectable TgAb (106). In theory, the higher the concentration of TgAb, the stronger its influence on Tg level, but this theory is not absolute. In some cases, low-concentration TgAb can be accompanied by a strong interference, which means that, sometimes, the qualitative characteristics of TgAb are associated with whether the interference actually occurs rather than the concentration (107). Positive TgAb is related to continuous antigenic stimulation and reflects immune system activity (108). Okosieme et al. (109) found that TgAb in patients with DTC had restricted and broad specificities. Antibodies with restricted specificities might result in a higher interference, which were mainly for analytical binding sites. However, antibodies with broad specificities are able to bind with different epitopes and can thereby be associated with lower interference. A typical interference performance of TgAb is the different Tg levels produced between RIA and IMA measurements (98). Compared with RIA measurements, IMA shows more low or undetectable Tg in the presence of TgAb (110). Alternatively, Tg measured by RIA could be used for most patients with positive TgAb as RIA measurements are less affected by TgAb (101). However, Crane et al. (105) stated that TgAb interfered with Tg measurements regardless of whether IMA or RIA measurement was used. They suggested that IMA and RIA measurements could be used in combination to detect Tg levels. In addition, LC-MS/MS is gradually becoming regarded as an effective tool to detect Tg in the presence of TgAb. Kushnir et al. (102) verified that MS could identify undetected Tg by immunoassay in the presence of TgAb. Similarly, Netzel et al. (101) found that 22% of cases with positive TgAb had detectable Tg by MS, but not by immunoassay. For the moment, LC-MS/MS appears to be the most suitable test for Tg detection in the presence of positive TgAb. However, the analytical sensitivity of LC-MS/MS was inferior and it could not detect Tg in patients with the structural disease (104).

In summary, each measurement for detecting Tg has its advantages and disadvantages. There are no clear rules regarding which measurement should be used for Tg in the clinical setting. Furthermore, it may be preferable for each patient to be continuously monitored using the same method. Compared with the interference of TgAb with serum Tg, the interference of serum TgAb with FNA-Tg seems to be insignificant. In follow-up patients, FNA-Tg was verified as a useful test, independently of TgAb (111). However, Jeon et al. (112) also found that excessive levels of serum TgAb might reduce FNA-Tg level by interfering with its measurement.

## 5 Conclusion

Researchers and clinicians have recently paid more attention to the role of Tg in DTC. It is known that malignant cells in DTC are well-differentiated and close to normal follicular epithelial cells; they retain the functions of iodine uptake, TSH stimulation, and Tg secretion. Therefore, Tg can be viewed as a tumor marker in DTC, but not in medullary and undifferentiated thyroid carcinoma. Current research suggests that Tg may have some effect in the diagnosis of malignant TNs before surgery. Preoperatively, normal thyroid tissue can also secrete Tg. In addition to normal tissue, when the patients have acute thyroiditis, thyroid cells are destroyed and stored Tg is released, which increases Tg serum levels. Therefore, it is difficult to define “elevated Tg”. The role of Tg in preoperative evaluation of patients with DTC needs further study. Postoperatively, elevated Tg is associated with a poor prognosis, and it is instrumental in guiding the treatment of RAI ablation. Nevertheless, the level of FNA-Tg plays a notable role in CLN metastasis whether preoperatively or postoperatively. Owing to the interference of TgAb, the Tg level often does not reflect the true situation and has limited application. In order to solve the problem, LC-MS/MS and other novel detection methods are gradually being applied in Tg clinical measurement; however, more studies are needed to convincingly demonstrate their clinical applicability.

## Author Contributions

SL, CR, and YG conceived and designed the study. Administrative support was provided by YG and JH. Study materials were provided by FY, JX, YT, and CG, who collected information and assembled the reviews. Review analysis and interpretation were performed by SL, CR, and FY. All authors contributed to the article and approved the submitted version.

## Funding

This work was supported by the National Key R&D Program of China under Grant 2019YFE0190500.

## Conflict of Interest

The authors declare that the research was conducted in the absence of any commercial or financial relationships that could be construed as a potential conflict of interest.

## Publisher’s Note

All claims expressed in this article are solely those of the authors and do not necessarily represent those of their affiliated organizations, or those of the publisher, the editors and the reviewers. Any product that may be evaluated in this article, or claim that may be made by its manufacturer, is not guaranteed or endorsed by the publisher.
